# Numerical Analysis of Steady-State Multi-Field Coupling in Electro-Fused Magnesia Furnace

**DOI:** 10.3390/ma18051049

**Published:** 2025-02-27

**Authors:** Cunjian Weng, Zhen Wang, Xianping Luo, Hui Li

**Affiliations:** 1 College of Materials Science and Engineering, Xi’an University of Architecture and Technology, No. 13 Yanta Road, Xi’an 710055, China; 2 Qinghai Western Magnesium Co., Ltd., Weiqilu Industrial Zone, Delingha 817000, China; 3 School of Electrical Engineering, Dalian University of Technology, No. 2 Linggong Road, Ganjingzi District, Dalian 116024, China; 4 School of Resources and Environmental Engineering, Jiangxi University of Science and Technology, No. 86, Hongqi Ave., Ganzhou 341000, China; 5 School of Water Resources and Environmental Engineering, East China University of Technology, No. 418, Guanglan Ave., Nanchang 330013, China

**Keywords:** MgO, numerical simulation, electric arc furnace, multi-physics modeling

## Abstract

The internal conditions of the high-temperature molten pool in an electro-fused magnesia furnace (EFMF) are difficult to measure, and the temperature distribution–energy conservation relationship in the EFMF cannot be effectively evaluated. Assuming that the feeding speed is constant, the heat absorbed by the newly added raw materials is equal to the rated power minus the heating power required to maintain thermal balance. Therefore, the EFMF can be approximately described by a steady-state model. In order to analyze the state of the molten pool of EFMF at different smelting stages, this study first constructed a three-dimensional steady-state multi-physics field numerical simulation model. The calculations show that the equivalent resistance of the molten pool varies approximately between 1 mΩ and 0.4 mΩ. Furthermore, the equivalent reactance produced by the whole conductive circuit is almost of the same order as the resistance. The Reynolds number of the convection inside the molten pool exceeds $10^{5}$, which means that the flow inside the molten pool is forced convection dominated by the Lorentz force. Moreover, the turbulence makes the temperature uniformity of the molten pool (the temperature gradient near the solid–liquid interface is approximately within 300 K/m) far greater than that of the unmelted raw materials with very low thermal conductivity (the average temperature gradient reaches over 1000 K/m); the respective proportions of arc power and Joule heating power can be predicted by the model. When the molten pool size is small, the proportion of Joule heating power is high, reaching about 20% of the rated power (3700 kVA); as the molten pool size increases, the convection effect is relatively weakened, and the proportion of Joule heating power also decreases accordingly, only 5% to 10%; the model prediction and experimental estimation results are in good agreement, which makes it feasible to conduct a quantitative analysis of the power distribution in different smelting stages.

## 1. Introduction

Electric arc smelting of magnesia involves melting magnesite (MgCO_3_) or magnesia (MgO) in an electric arc furnace and then solidifying the MgO. Due to the significant differences in the smelting process compared to steelmaking, this type of electric arc furnace is often referred to as an electro-fused magnesia furnace (EFMF). Products made from fused magnesia have a wide range of industrial applications due to their unique physical and chemical properties. From a mineralogical perspective, its mineral composition is mainly periclase, which exhibits an isometric crystal system structure [1,2]. Pure periclase is colorless, but as the content of impurities increases, its color gradually deepens, showing yellow, brown, or dark-brown hues. In addition, as the calcination temperature rises and the holding time prolongs, the grain size of periclase gradually increases, and its resistance to hydration and slag erosion is enhanced [3,4]. Their outstanding high-temperature resistance and thermal stability render them indispensable materials for furnace lining in the steelmaking process [5]. Moreover, due to their remarkable high-temperature resistance and excellent light transmittance, MgO single crystals in high-purity fused magnesia products are utilized as high-temperature optical materials in the aerospace field [6].

During the melting process in the EFMF, there are various harsh factors such as spatial enclosure, high melting points of raw materials, high dust concentration, and strong electromagnetic interference, which make it technically infeasible to monitor the internal raw material temperature field. To overcome the difficulties in monitoring, many scholars have adopted advanced control theories and artificial intelligence technologies to study its processes, including the recognition and diagnosis of abnormal states or faults [7,8,9], intelligent evaluation of operational performance [10], and design of stable control rules for three-phase currents [11,12]. However, research in these areas has not paid much attention to the physical processes inside the EFMF. The physical processes involved are governed by a multitude of factors, including electromagnetic fields, fluid dynamics, temperature gradients, and phase transitions between solids, liquids, and gases. The intricate interplay among these factors significantly aids in elucidating the complex smelting processes that are typically challenging to monitor.

Arc fusion can process a wide variety of non-metallic minerals, and the related smelting equipment is collectively referred to as a submerged arc furnace (SAF). In recent years, the study of smelting processes in SAFs using multi-field coupling numerical simulation technology has gradually received widespread attention. Zhang Xuankai et al. [13,14] established a transient three-dimensional (3D) mathematical model to analyze the distribution of multi-physical fields such as electromagnetic, temperature, and composition during the smelting process of calcium carbide (${\mathrm{CaC}}_{2}$). The model reveals that there is strong coupling and non-uniformity among the multi-physical fields, and large-capacity SAFs have significant qualified advantages in increasing production. Henan Cui et al. [15] developed a transient three-dimensional mathematical model to analyze the speed–temperature–electromagnetic field in the smelting reduction process of titanic iron ore in an electric arc furnace (EAF), concluding that the Lorentz force is the main driving force for the distribution of speed and temperature; however, the model did not explain why, under the action of three-phase balanced AC power, the titanic slag and molten iron would show a counterclockwise circulation in the horizontal direction. Li Baokuan et al. [16] aimed to study the selection and control of operational parameters for titanic slag EAFs; they established a three-dimensional multi-physical field model for the transmission and reaction process of materials, titanic slag, and iron water, and the model also ignored the forced convection phenomenon caused by high current. Karalis et al. [17,18] established a model for the smelting of nickel–iron alloy in a three-phase EAF. Although the model calculates the flow field and judges the molten slag as laminar flow based on the Reynolds number, it does not provide a detailed analysis of the various influencing factors of this convective phenomenon. Tesfahunegn et al. analyzed the induced skin and proximity effects of the SAF using numerical simulation methods. They discovered that, by sacrificing a certain degree of calculation accuracy, the AC solver could be replaced with a DC solver to achieve higher computational efficiency [19].

Multi-physical field coupling numerical simulation remains one of the most effective methods to estimate various physical fields inside the EFMFs. Tianchi Jiang et al. [20] developed a 3D transient multi-physical field model to capture phenomena such as electromagnetism, thermodynamics, decomposition reactions, and flow within the EFMF. However, the model did not delve into the morphology of the molten pool and its mutual influences with the multi-physical fields. Wang et al. [21,22] conducted a numerical simulation analysis of the operation of a 3000 kVA AC EFMF, revealing the distribution of the flow and temperature fields of the molten pool under electromagnetic stirring. It was found that there is a significant temperature gradient on both sides of the phase change interface between the graphite electrode and the furnace wall, and the phase change interface in this area is more stable compared to other areas; thus, larger crystals are more likely to appear at the bottom of the molten pool in this location.

In order to estimate the heating effect of the electric arc on the molten pool, they also conducted a numerical simulation analysis of the electric arc plasma in a double electrode DC EAF for magnesia smelting, successfully obtaining the distribution of the electric field, magnetic field, temperature field, flow field, and pressure field of the electric arc under different arc lengths. Based on this, the four heat transfer mechanisms of the electric arc (convection heat transfer, radiation heat transfer, Thomson effect, and electron condensation) on the surface of the molten pool, as well as the shape of the molten pool when it reaches a stable state, were calculated [23]. The morphology of the arc plasma within the EFMF and its interaction with the molten pool are almost unobservable during the production process. Therefore, the conclusions of the above-mentioned model are difficult to verify with experimental data. DC arc furnaces possess advantages such as higher heating power, better stability, a higher power factor, and lower electrode consumption. To facilitate the formation of conductive channels, Wang et al. also studied the multi-physical field distribution within a dual-electrode DC EFMF. They found that when the effective current values of the DC furnace and the AC furnace are the same, the electromagnetic stirring intensity and the distribution of Joule heating power are quite similar. The main difference lies in the heating power value generated by the arcs [24,25].

Combined with on-site research on the smelting process, it is found that, when producing fused magnesia products from high-purity raw materials, the crucial links for improving the yield and quality are whether impurity removal and crystal growth can be well-controlled. However, the input power during smelting always approaches the rated power, far exceeding the thermal balance heating power required for impurity segregation and crystal growth. In order to quantitatively analyze the relationships among various power values under thermal balance, we propose a steady-state multi-physical field analysis model. This model aims to clarify the reasonable range of the input power and its process principles, so as to provide a theoretical basis for impurity removal and crystal growth. First, we analyze the electromagnetic field distribution and simplify the results with a lumped parameter circuit model to uncover the impedance characteristics related to the molten pool’s morphology. Based on the time-harmonic electromagnetic field analysis, we examine the non-isothermal flow of the molten pool under electromagnetic stirring and the thermal balance influenced by arc heating and Joule heating. After calculating the temperature field of the molten pool and unmelted raw materials, we analyze the convection and radiation heat dissipation power for different molten pool sizes. We also explore the dimensionless numbers of the melt convection in the pool.

## 2. Experimental Procedure

**Figure 1:**
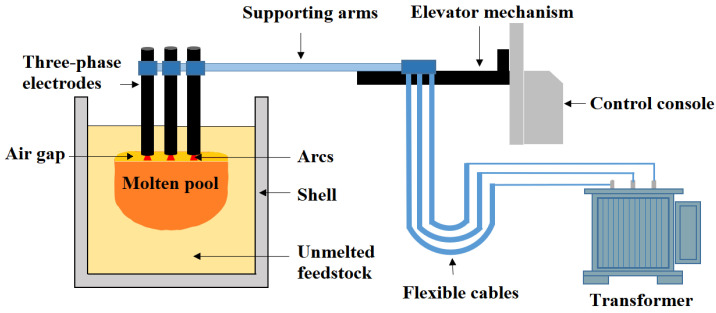
Schematic diagram of EFMF: The figure illustrates the basic components of the EFMF system, including the power supply, electrode lifting system, electrodes, and furnace body. It highlights the fundamental operation process and the molten pool’s state, providing a clear overview of the system’s layout and functionality.

The AC EFMF system mainly consists of the following components: a power supply system (including a monitoring system and a transformer), an electrode lifting and control system, flexible cables, supporting arms, electrodes, and the furnace body itself (shown in Figure 1). The experimental EFMF is equipped with a transformer with a capacity of 3700 kVA. The furnace shell has a diameter of 3 m and a height of 3.2 m. Each graphite electrode has a diameter of 0.4 m, and the distance between electrodes is 0.75 m.

The experimental procedure is primarily segmented into several key stages: the arc ignition phase (characterized by a small-scale molten pool), the molten pool formation phase (with a medium-scale molten pool), the automatic smelting phase (involving a large-scale molten pool), the furnace shutdown and heat preservation phase, and finally the crushing and decomposition phase. The critical operational steps encompass the following:

(1) First, lay a 600 mm thick layer of raw materials at the bottom of the EFMF, and then continuously add the raw materials until the graphite electrodes are buried to a depth of 500 mm.

(2) Check whether the cooling water circulation system, hydraulic system, transformer, etc., are functioning properly, and then activate the switch to initiate the smelting process.

(3) During the arc ignition phase, the current is maintained at 10,000 A (with a tolerance of ±500 A), and the secondary voltage of the transformer is set to the 136 V tap position.

(4) During the intermediate and later stages, the current is regulated at 14,000 A to 18,000 A, and the secondary voltage of the transformer is adjusted to the 152 V setting.

(5) The feeding frequency is 6 to 8 times per hour, and the feeding depth is managed to approximately 150 mm.

(6) A total of 4 smelting experiments were carried out, and the cumulative feeding weight in each experiment reached 8 to 10 tons.

(7) The electric melting duration exceeds 8 h.

(8) The cooling period spans approximately 7 to 8 days.

## 3. Modeling

Before performing steady-state analysis, the following assumptions are also needed:

(1) The AC power is in a balanced three-phase state, and only the fundamental frequency (i.e., line frequency) component is present, ignoring higher harmonic components.

(2) When the arc power is maintained stable, the gas generated by the raw materials will form a stable air interlayer between the molten pool and the unmelted raw materials, preventing the unmelted raw materials from mixing into the molten pool.

(3) The raw materials are in solid or liquid states, and the gaseous components in the raw materials are not considered.

(4) The heating of raw materials by electric arcs is described by a Gaussian distribution of volumetric heat sources.

(5) Ignore the effects of the ablation and the slow lifting process of graphite electrodes on the results of the steady-state analysis. Ablation is a process in which the electrodes rise slowly. The electrodes can be controlled by the lifting device to descend slowly. The effects of these two processes counteract each other, thus stabilizing the current and voltage values required for thermal balance.

(6) Ignore the effects of components far away such as the furnace shell, furnace transport vehicle, supporting arms, and water-cooled cables on the calculation results (proven to have minimal impact on the molten pool and its surrounding areas [26]).

**Figure 2:**
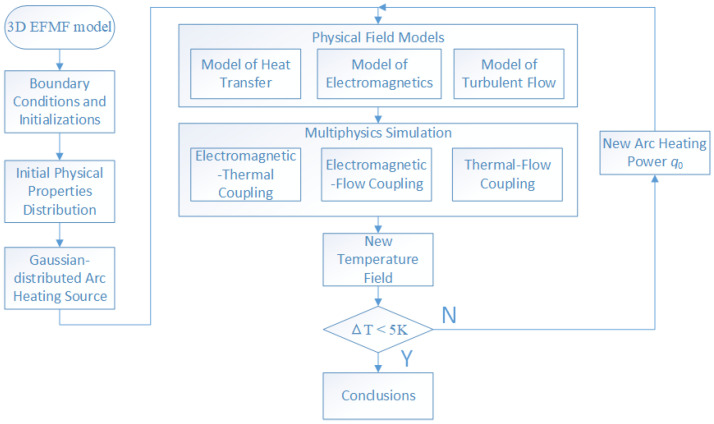
The flowchart of a multi-physics simulation process for the EFMF: The flowchart outlines the multi-physics simulation process for the EFMF, highlighting the sequential steps to achieve a steady state. It streamlines the simulation setup, ensuring accurate and efficient convergence to steady-state conditions.

Based on the above-mentioned considerations, the numerical simulation process of multi-physics fields (electromagnetic, thermal, and fluid) for EFMF is shown in Figure 2. First, a 3D model is constructed, the boundary conditions of the model are determined, and initialization is carried out. At the same time, the initial distribution of physical properties is clarified, and a Gaussian-distributed arc heating source is introduced. Next, physical field models are established, covering the heat transfer model, the electromagnetic model, and the turbulent flow model. Subsequently, multi-physics field simulations are carried out to achieve electromagnetic–thermal coupling, electromagnetic–fluid coupling, and thermal–fluid coupling. The simulation will generate a new temperature field, and the temperature difference between the temperature at the solid–liquid interface and the melting point is judged. If the temperature difference is greater than the set threshold (ΔT > 5 K), a new arc heating power $q_{0}$ is determined; if this condition is met (ΔT < 5 K), simulation conclusions are drawn.

### 3.1. Electromagnetic Field Model

Electromagnetic field analysis for the EFMF is considered in three aspects. First, the alternating current will induce eddy currents in surrounding conductive and magnetic materials, leading to losses. Second, the power consumed by various components in the conductive circuit of the EFMF has certain guiding significance for the setting of control parameters and the design of energy-saving and consumption reduction. Third, the Lorentz force and Joule heat generated by the alternating current play a controlling role in the convection and temperature field distribution within the molten pool, forming the basis for subsequent multi-field coupling analysis.

#### Governing Equations, Material Properties, and Boundary Conditions

The Maxwell’s equations involved in time-harmonic electromagnetic fields include Ampere’s law, Faraday’s law of electromagnetic induction, and Gauss’s law for magnetism. Since only a low-frequency 50 Hz alternating current is involved, the displacement current term can be neglected, and they can be further simplified into two partial differential equations containing only scalar electric potential and vector magnetic potential. With the addition of boundary conditions, they can be solved using the finite element method. The relevant governing equations are shown as follows [13,21,27]:

Faraday’s law of electromagnetic induction:

(1) $$\nabla \times \dot{E}=-j\omega \dot{B}$$

Gauss’s law for magnetic fields:

(2) $$\nabla \cdot \dot{B}=0$$

Ampere’s law:

(3) $$\nabla \times \dot{H}=\dot{J}+j\omega \dot{D}$$

where $\dot{H}$ is the magnetic field strength, $\dot{J}$ is the current density, $\dot{D}$ is the electric displacement, $\dot{E}$ is the electric field intensity, $\dot{B}$ is the magnetic flux density, and ω is angular frequency.

The constitutive equations for the electromagnetic fields are as follows:

(4) $$\dot{B}=\mu \dot{H}$$

(5) $$\dot{J}=\sigma \dot{E}$$

where μ is the magnetic permeability, and σ is the electrical conductivity.

The EFMF mainly consists of components such as transformer, bus bars, water-cooled cables, supporting arms, lifting mechanisms, graphite electrodes, clamping mechanisms, furnace body, and furnace dolly. To simplify the calculation, only the graphite electrode and the heated raw material parts are retained. The physical parameters of molten MgO and the size parameters of the EFMF are shown in Table 1. The molten pool is divided into three sizes: large, medium, and small. The long diameter is consistent and the depth shows a more significant change. The corresponding variables are shown in Table 2. The choices of dimensional parameters of EMFE for different scales were obtained by averaging the measurement and statistical results of the morphologies of solidified ingots based on multiple on-site experiments.

**Figure 3:**
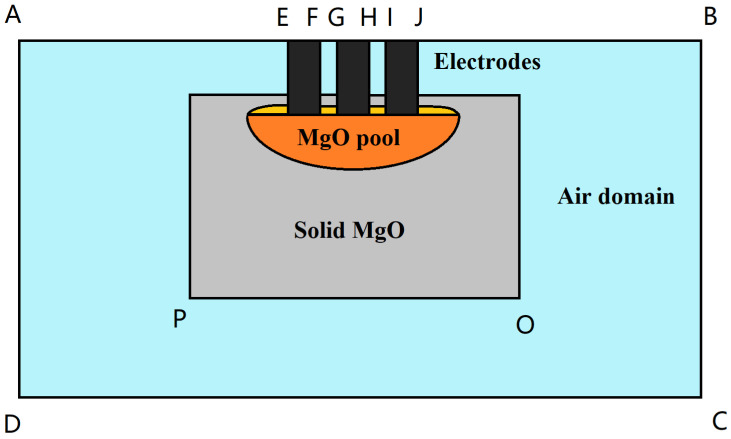
Schematic diagram of calculation domain and boundary conditions for the EFMF (electro-fused magnesia furnace): The diagram illustrates the computational domain and boundary conditions for the electromagnetic field, providing a clear overview of the setup and facilitating accurate simulation.

According to the uniqueness theorem of electromagnetic fields, to determine the distribution of electromagnetic fields, including scalar potentials and vector magnetic potentials, boundary conditions must also be provided. The boundaries involved in the boundary conditions are shown in Figure 3. The outer boundary of the air domain, except for the upper surface (i.e., including only AD, BC, and CB), is set to be magnetically insulated; the outer boundary of the air domain, except for EF, GH, and IJ, is set to be electrically insulated. IJ, GH, and EF are, respectively, set as the current input terminals for the three phases of alternating current with a phase difference of 120 degrees.

### 3.2. Temperature and Flow Field Model

#### Governing Equations, Material Properties, and Boundary Conditions

The macroscopic transport phenomena in the EFMF are governed by the conservation equations of mass, momentum, and energy in the solid and liquid phases, involving the following governing equations.

A statement of the conservation of mass is given in the following form:

(6) $$\nabla \cdot \left(\rho v\right)=0$$

where ρ is the density of the melt, and v is the velocity of the melt. In an inertial frame of reference, the conservation form of the equations of continuum motion is:

(7) $$\nabla \cdot \left(\rho vv\right)=-\nabla \cdot pI+\nabla \cdot \left[\mu \left(\nabla v+\nabla v^{T}\right)\right]+\rho g+f_{L}$$

where *p* is the pressure, I is the identity matrix, g is acceleration of gravity, and $f_{L}$ is the Lorentz force.

Conservation of energy:

(8) $$\nabla \cdot \left(\rho vT\right)=\nabla \cdot \left(\frac{\lambda }{C_{p}}\nabla T\right)+Q$$

where λ is the thermal conductivity, $C_{p}$ is the specific heat, *Q* is the volumetric heat source, and *T* is the temperature. *Q* is composed of two parts.

(9) $$Q=Q_{Joule}+Q_{arcs}=\frac{{\left|\dot{J}\right|}^{2}}{\sigma }+\frac{6\sqrt{3}q_{0}}{\pi \sqrt{\pi }R_{e}^{3}}e^{-\frac{3r^{2}}{R_{e}^{2}}}$$

where $Q_{Joule}$ is the Joule heating source, $Q_{arcs}$ is the arc heating source, $q_{0}$ is the total power released by the heat source in the 3D space, and $R_{e}$ is the radius of electrodes.

The flow was solved using the k−ε turbulence model, and the turbulent kinetic energy equation is described as follows [23,30]:

(10) $$\nabla \cdot \left(\rho vk\right)=\nabla \cdot \left(\left(\mu +\frac{\mu_{t}}{\sigma_{k}}\right)\nabla k\right)-\rho \varepsilon +G_{k}$$

where *k* represents the turbulence kinetic energy, ε represents its dissipation rate, and $G_{k}$ represents the generation of turbulence kinetic energy due to the mean velocity gradients. The turbulent (or eddy) viscosity, $\mu_{t}$, is computed by combining *k* and ε, as follows:

(11) $$\mu_{t}=\rho C_{\mu }k^{2}/\varepsilon$$

The dissipation rate equation is:

(12) $$\nabla \cdot \left(\rho v\varepsilon \right)=\nabla \cdot \left(\left(\mu +\frac{\mu_{t}}{\sigma_{\varepsilon }}\right)\nabla \varepsilon \right)+\frac{\varepsilon }{k}\left(C_{1\varepsilon }G_{k}-C_{2\varepsilon }\rho \varepsilon \right)$$

where the model constants have the following default values: $C_{1\varepsilon }$ = 1.44, $C_{2\varepsilon }$ = 1.92, $C_{\mu }$ = 0.09, $\sigma_{k}$ = 1.0, and $\sigma_{\varepsilon }$ = 1.3.

The material properties of molten MgO and the dimensional parameters of the EFMF are shown in Table 1. The molten pool is divided into three cases: small, medium, and large. The small melting pool corresponds to the initial stage of smelting, where the pool volume is small, the depth is shallow, and the length and width diameters of the pool are also small, not fully spread out. The medium melting pool corresponds to the middle stage of smelting, where the pool has reached a certain scale and has a certain depth, and the length and width diameters of the pool have reached a certain length, basically meeting the conditions for charging, and can continue to develop in depth. The large melting pool corresponds to the later stage of smelting, where the size of the pool has basically formed, the depth has reached the deepest, and it begins to prepare for solidification. The relevant physical properties of MgO are shown in Table 3. The raw material is surrounded by a furnace shell made of stainless steel. Based on experience, the convective heat dissipation coefficient (*h*) of its surface can be set to 10 W/(m^2^·K), and the radiative emissivity (ϵ) of its surface is 0.8 [31]. In this model, the furnace shell is not modeled, and it is assumed that the influence of the furnace shell can be ignored. Therefore, the two coefficients are directly set on the surface of the MgO raw material.

**Figure 4:**
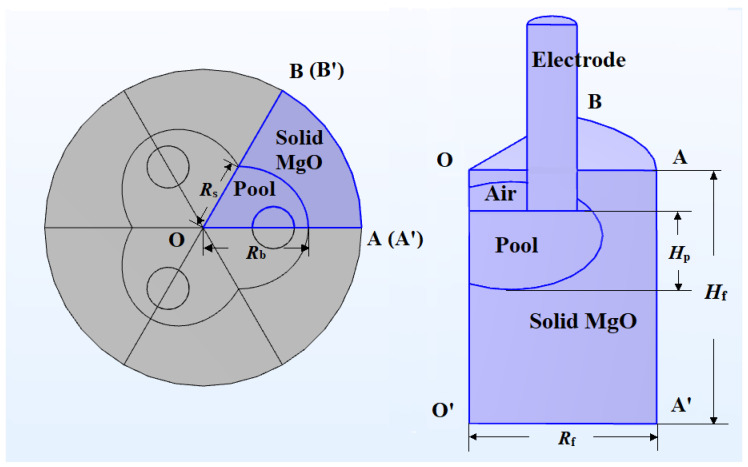
The top view and front view of the 1/6 simplified model: The figure shows the top and front views of the 1/6 simplified model, highlighting its symmetrical configuration. This visualization illustrates how the temperature and flow field calculations are simplified using the symmetrical 1/6 model, enhancing computational efficiency.

To simplify the calculations, based on the assumption that the three-phase alternating current is symmetrical in space, the model is considered to be in a 1/6 symmetrical situation, so only 1/6 of the model is taken for calculation, as shown in Figure 4. On the basis of the 1/6 model, boundary conditions are set as follows: surfaces O$O^{\prime }$$A^{\prime }$A and O$O^{\prime }$$B^{\prime }$B are symmetrical surfaces, $O^{\prime }$$A^{\prime }$$B^{\prime }$, OAB, and A$A^{\prime }$$B^{\prime }$B are surfaces for convective and radiative heat dissipation, and the bottom area of the graphite electrode is the equivalent heat flux of arc heating. The convective heat transfer coefficient and the emissivity of thermal radiation are 10 W/($m^{2}$·K) and 0.8, respectively, with the ambient temperature set to 300 K.

## 4. Results and Discussion

### 4.1. Analysis of Electromagnetic Fields

**Figure 5:**
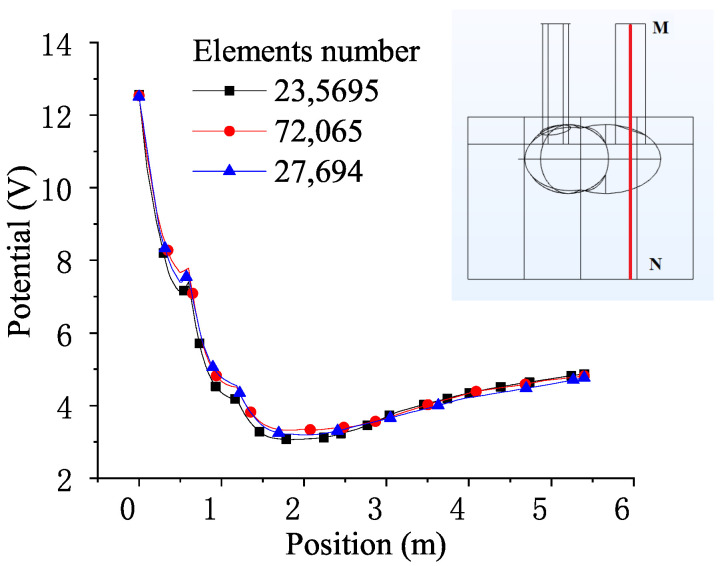
Mesh sensitivity analysis and the location of the corresponding sampling points: Mesh sensitivity analysis shows that the electric field distribution stabilizes when the grid number exceeds 20,000. The analysis determines the appropriate mesh number and thus prevents the unnecessary consumption of excessive computational resources.

Figure 5 shows the variation in the electric potential vertically downward from the center of the zero-phase electrode (from point M to point N) as the distance increases. Since the currents flowing into the three graphite electrodes differ in phase by 120 degrees from each other (0°, 120°, and −120°), to make a distinction, the electrode on the plane OA$A^{\prime }$$O^{\prime }$ is set to have a phase of 0° shown in Figure 5. By observing the grid sensitivity pattern with respect to grid variation, it can be seen that a significant increase in grid density does not lead to a substantial change in the calculation results. In other words, the calculation of the electric field distribution is not significantly related to the grid number when the grid quantity is above 20,000.

**Figure 6:**
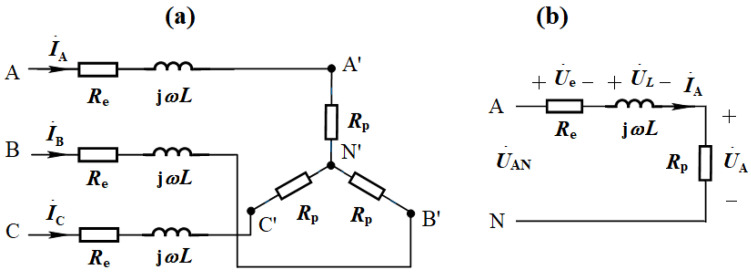
Equivalent circuit diagram of molten pool and the electrodes: (**a**) star-connected load; (**b**) an equivalent single-phase circuit. In a three-phase balanced state, the molten pool and electrodes are simplified as a star-connected load and represented by an equivalent single-phase circuit. This diagram simplifies the understanding of key electrical concepts (voltage, current, power) for the molten pool and electrodes, facilitating discussions in subsequent sections.

The three-phase alternating current is set to have a phase difference of 120 degrees, with an effective value of 14,000 A. The equivalent circuit of the molten pool is depicted in Figure 6a. ${\dot{U}}_{A}$ and ${\dot{I}}_{A}$ are the equivalent phase voltage and current of the molten pool in phase A, respectively, and $U_{A}$ and $I_{A}$ are their effective values. Given that the smelting process is assumed to be in a three-phase balanced state, the three-phase circuit can be equivalent to a single-phase circuit, as shown in Figure 6b. $R_{el}$, ${\dot{U}}_{e}$, $P_{e}$, *L*, ${\dot{U}}_{L}$, $R_{p}$, ${\dot{U}}_{AN}$, and $P_{melt}$ represent the equivalent resistance of one graphite electrode, the voltage drop across this electrode, the Joule heating power of all electrodes, the equivalent inductance, the voltage drop across the inductance, the equivalent resistance of the molten pool, the equivalent phase voltage of all the above-mentioned loads, and the active power (Joule heating power) of the entire molten pool, respectively. In this model, the impedance characteristics of the arc are not taken into account due to the strong randomness and nonlinearity of the arc’s volt–ampere relationship. Instead, the impedance characteristics of the arc itself are inferred from the voltage drops and currents in the other components of the circuit.

The resistance of the graphite electrode remains constant, calculated by the formula Re=ρl/S, approximately 0.122 mΩ. The corresponding voltage drop $U_{e}$ is about 1.7 V, and the corresponding power $P_{e}$ is 71.5 kW. If the molten pool is considered as a star-connected load, the changes of $R_{p}$, jωL, $U_{A}$, and $P_{melt}$ are as shown in Table 4. Assuming $I_{A}$ = 14,000 A, the $U_{A}$ would vary approximately between 6.19 V and 14.35 V, and $P_{melt}$ would vary approximately between 256 kW and 603 kW. The shape and size of the molten pool have little effect on the equivalent inductance in the circuit, the inductive reactance is less than the resistance of the molten pool, and is of the same order of magnitude as the resistance of the molten pool.

**Figure 7:**
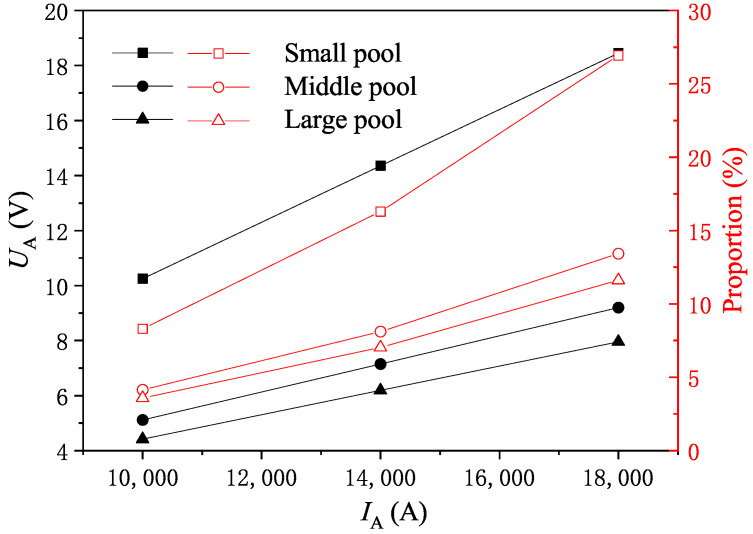
Variation in phase voltage $U_{A}$ and $P_{melt}/P_{t}$ with phase current $I_{A}$: The figure shows the linear relationship between phase voltage $U_{A}$ and phase current $I_{A}$, and indicates that the proportion of Joule heating power ($P_{melt}$) in the total power ($P_{t}$) is relatively small. This data highlight the importance of simulation and modeling for optimizing the smelting process, as critical parameters like voltage drop and absorbed Joule heating power are typically not directly measurable during operation.

The variation in $U_{A}$ and the proportion of $P_{melt}$ absorbed by differently sized molten pools to the total input active power $P_{t}$ is shown in Figure 7. The phase voltage and phase current of the molten pool exhibit a linear relationship. When the molten pool is relatively small in size and $I_{A}$ exceeds 10,000 A, $U_{A}$ will exceed 10 V. However, if the molten pool reaches a medium or larger size, the voltage will drop significantly; here, even when $I_{A}$ reaches 18,000 A, $U_{A}$ will not exceed 10 V. With $I_{A}$ remaining constant, the size of the molten pool significantly influences the proportion of its Joule heating power relative to the rated power. A small-scale molten pool can account for nearly 30% of the thermal power consumption, whereas a larger-scale molten pool contributes to less than 10%. $P_{t}$ is determined by the transformer and is about 3700 kW.

**Figure 8:**
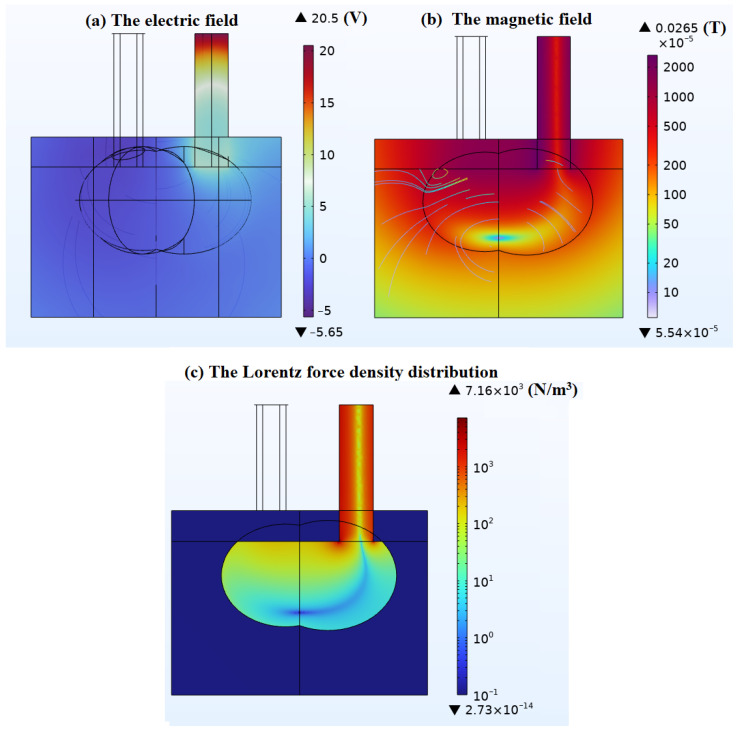
Analysis of electromagnetic field calculation results when $I_{A}$ = 14,000 A: (**a**) the electric field (V); (**b**) the magnetic field (T) in logarithmic coordinate system; (**c**) the Lorentz force density distributions (N/m^3^) in logarithmic coordinate system. The results show the electric field distribution, magnetic field distribution, and Lorentz force density under a constant current of 14,000 A, highlighting their dependence on molten pool size and current density. The above findings indicate that the Lorentz force is the primary driving force for melt convection, causing the turbulence phenomena discussed later.

When $I_{A}$ = 14,000 A, the electric and magnetic field distributions, as well as the Lorentz force, are illustrated in Figure 8. The electric field distribution confirms that the electric field strength is directly proportional to the current magnitude; when the current is held constant, the smaller the molten pool, the greater the electric field intensity. The magnetic field distribution reveals that the magnetic flux density is lower near the bottom of the molten pool and higher at the surface. The Lorentz force is directed from the exterior of the conductor towards its interior, encompassing both the graphite electrodes and the molten pool. It is evident that the average Lorentz force density within the graphite electrodes is relatively high due to the concentrated distribution of current density within the electrodes. In contrast, within the molten pool, aside from the vicinity of the graphite electrode’s base, the Lorentz force density is more dispersed, a result of the tendency for current density to spread out within the molten pool. Theoretically, the Lorentz force density varies with the excitation current in a quadratic relationship. For instance, if the excitation current is doubled, both the current density and magnetic flux density distributions will also double, leading to a quadrupling of the overall Lorentz force density. The Lorentz force density is also significantly influenced by the shape of the molten pool. A larger molten pool volume results in a more dispersed current density and, consequently, a more dispersed Lorentz force density; conversely, a smaller molten pool volume leads to a more concentrated current density and a more concentrated Lorentz force density. From these observations, it can be deduced that in the early stages of smelting, when the molten pool is smaller, the stirring intensity due to the Lorentz force under the same level of current excitation is significantly stronger than in the later stages of smelting.

### 4.2. Analysis of Temperature and Velocity Fields

**Figure 9:**
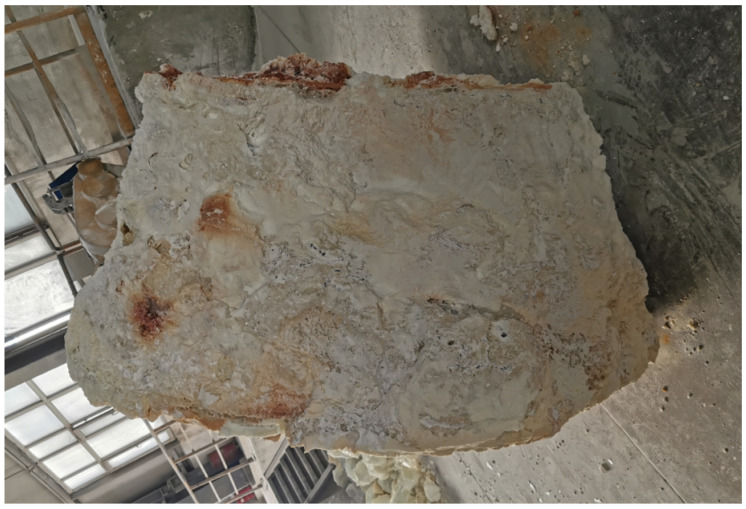
Shape of a MgO ingot (about 4 tons): The solidified MgO ingot has a pear-like shape, wider at the base and tapering towards the top. Although the molten pool in the smelting experiment cannot be directly measured, the smelting process can be inferred from the shape and structure of the solidified MgO ingot.

The flow field within the EFMF has been established in the existing literature [21,24] to exhibit a significant Lorentz force stirring effect under currents exceeding tens of thousands of amperes. The stirring effect facilitates forced convection within the molten pool, thereby substantially enhancing the temperature uniformity across the pool. Given that the raw materials used in these furnaces are of exceptional purity, often exceeding 99%, they can be treated as a homogeneous substance for practical purposes. Upon solidification, the morphology of the melt can be examined by allowing the ingot to cool completely, opening the furnace shell, and removing any residual unmelted MgO raw materials. The resulting MgO ingot typically exhibits a wider shape at the base and tapers towards the top, resembling a pear shown in Figure 9; while there is currently no dependable method to monitor the solid-liquid interface directly, the temperature and flow field distributions of the MgO melt throughout the smelting process can be effectively analyzed using multi-field coupling numerical simulations. To quantitatively assess the Joule heat and arc heat necessary for different balance states in the EFMF, the molten pool sizes are still categorized into three distinct categories: large, medium, and small. The Joule heating power is then determined based on $I_{A}$, which, according to actual smelting data, could be either 10,000 A or 14,000 A. Finally, the arc power is calculated by determining the thermal power required for the molten pool to achieve thermal balance.

#### 4.2.1. Small-Scale Molten Pool

**Figure 10:**
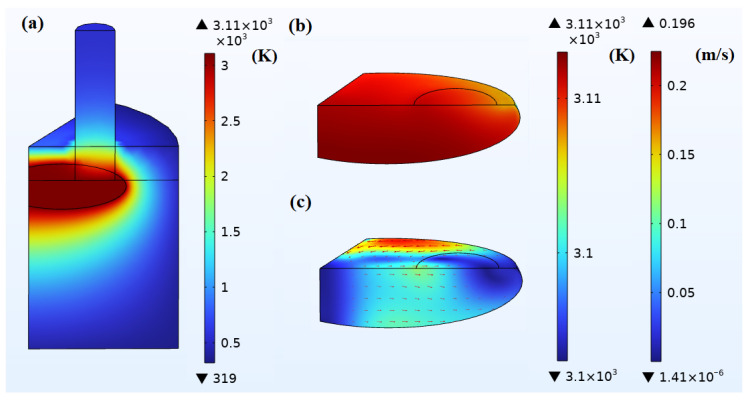
Simulation results of small-scale pool when $I_{A}$ = 10,000 A: (**a**) temperature field of the 1/6 model (K); (**b**) temperature field of the pool (K); (**c**) flow field of the pool (m/s). The simulation results show steady-state temperature and flow fields in the EFMF system and the small-scale molten pool when $I_{A}$ = 10,000 A, highlighting the steady-state conditions achieved under this current.

When the molten pool is maintained at a small scale and $I_{A}$ = 10,000 A, the 1/6 model and the internal molten pool achieve a steady-state temperature field and flow field as shown in Figure 10. $P_{melt}$ and $P_{e}$ are 309 kW and 42 kW, respectively. The total power released by the electric arcs at the three graphite electrodes $P_{arcs}$ is 36 kW. The total heating power released by the above three heat sources $P_{heating}$ is 387 kW. After calculation, although both arc and Joule heating power will vary individually, it is possible to achieve thermal balance for this small-scale molten pool in the EFMF by maintaining a constant total heating power. Under this condition, the heat dissipation power $Q_{dissipation}$ equals $P_{heating}$. Here, $I_{A}$ is not set to 14,000 A because this would result in the Joule heating power absorbed by the molten pool being as high as over 600 kW, far exceeding the required heating power of 387 kW for thermal balance, thus forming a larger molten pool, which will be discussed later. Under the thermal balance state, the power values of each item in the model satisfy the following relationship.

(13) $$P_{heating}=P_{melt}+P_{arcs}+P_{e}=Q_{dissipation}$$

Heat dissipation occurs in two ways: radiation and heat convection. The radiation heat dissipation of the graphite electrodes is as high as about 180 kW, and the radiation heat dissipation of the furnace walls and bottom is only about 120 kW, with convective heat dissipation from all external surfaces being about 80 kW. Observing the temperature field of the molten pool, it can be found that the temperature difference is about 15 K, while the temperature difference of the unmelted MgO is as high as about 2700 K. The overall temperature increase in the molten pool and the growth of the current basically follow a linear law. By observing the flow field of the molten pool, it can be found that the average flow velocity reaches about 0.1 m/s, the maximum flow velocity reaches 0.2 m/s, mainly in the horizontal direction. If observed from the top of the furnace, the melt forms a vortex flow mainly in a counterclockwise direction.

#### 4.2.2. Medium-Scale Molten Pool

**Figure 11:**
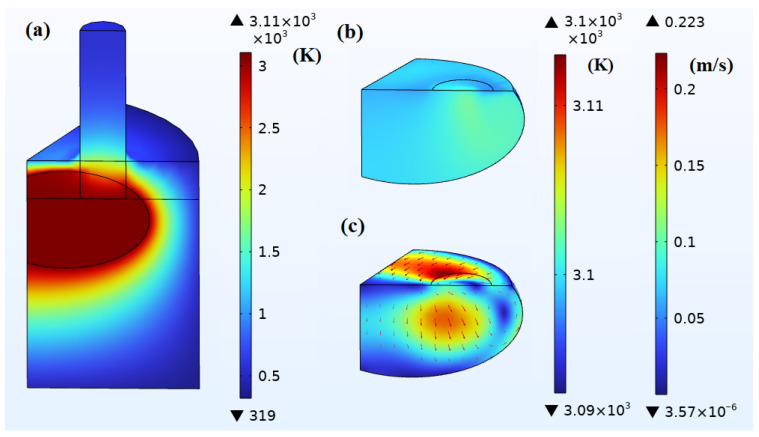
Simulation results of medium-scale pool when $I_{A}$ = 14,000 A: (**a**) temperature field of the 1/6 model (K); (**b**) temperature field of the pool (K); (**c**) flow field of the pool (m/s). The simulation results show steady-state temperature and flow fields in the EFMF system and the medium-scale molten pool when $I_{A}$ = 14,000 A, highlighting the steady-state conditions achieved under this current.

When the molten pool reaches a medium scale, the corresponding steady-state temperature field and flow field are shown in Figure 11, at which time $I_{A}$ = 14,000 A. $P_{melt}$ and $P_{e}$ are 304 kW and 79 kW, respectively, with a total that is essentially equal to that of the small molten pool. $P_{arcs}$ is 165 kW, which is approximately twice that of the small molten pool. $P_{heating}$ is 548 kW, meaning that, even though $I_{A}$ decreases and $P_{arcs}$ increases, the total power remains constant, allowing this molten pool to maintain thermal balance. $Q_{dissipation}$ also increases to 548 kW. Among it, the convective cooling power $Q_{conv}$ is 86 kW and the radiative cooling power $Q_{rad}$ is 462 kW. The temperature difference in the molten pool’s temperature field is about 9 K, as the strong electromagnetic stirring leads to improved temperature uniformity. The average velocity in the molten pool’s flow field reaches about 0.11 m/s, with the highest velocity at 0.22 m/s. Observing from the furnace wall near the zero-phase electrode into the furnace, the molten metal forms a vortex flow predominantly moving in a counterclockwise direction.

#### 4.2.3. Large-Scale Molten Pool

**Figure 12:**
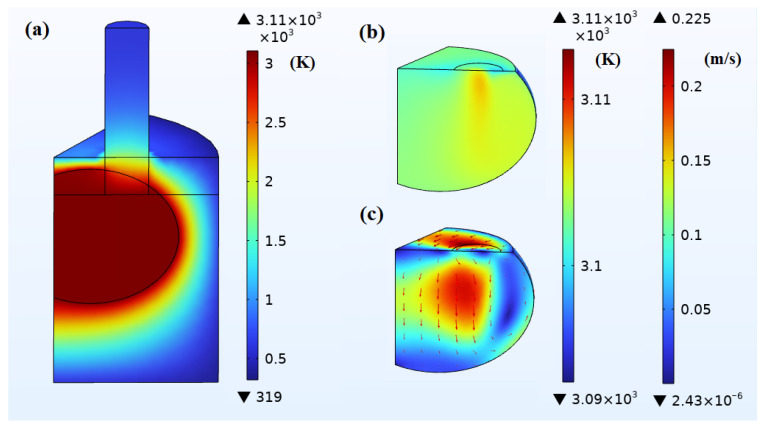
Simulation results of large-scale pool when $I_{A}$ = 14,000 A: (**a**) temperature field of the 1/6 model (K); (**b**) temperature field of the pool (K); (**c**) flow field of the pool (m/s). The simulation results show steady-state temperature and flow fields in the EFMF system and the large-scale molten pool when $I_{A}$ = 14,000 A, highlighting the steady-state conditions achieved under this current.

When the molten pool reaches a large scale, the corresponding steady-state temperature field and flow field are shown in Figure 12, at which time $I_{A}$ = 14,000 A. $P_{melt}$ and $P_{e}$ are 260 kW and 79 kW, respectively, with a total that is 44 kW lower than that of the medium-scale molten pool. $P_{arcs}$ is 252 kW, which is about 7 times that of the small molten pool and nearly 90 kW higher than that of the medium-scale molten pool. $P_{heating}$ is 591 kW, indicating a slight reduction in $P_{melt}$, while $P_{arcs}$ has increased significantly to maintain thermal balance. $Q_{dissipation}$ is composed of $Q_{conv}$, which is 124 kW, and $Q_{rad}$, which is 466 kW. The temperature difference in the molten pool’s temperature field is about 13 K, due to the significant increase in arc power leading to higher localized temperatures at the bottom of the electrodes. The average velocity in the molten pool’s flow field reaches about 0.11 m/s, with the highest velocity at 0.22 m/s, predominantly in the vertical direction. When observed from the top, the molten metal forms a vortex flow mainly moving in a counterclockwise direction. When viewed from the angle shown in the figure, a smaller counterclockwise flow vortex is also formed on the outside of the zero-phase electrode.

#### 4.2.4. Analysis of Dimensionless Numbers

To further analyze the characteristics of convection within the molten pool, dimensionless numbers such as the Reynolds, Grashof, Richardson, and Prandtl numbers were extracted for the three scenarios mentioned above (shown in Table 5). ρ, μ, α, and λ represent, respectively, the density, viscosity, thermal expansion rate, and thermal conductivity of the MgO melt. The values of these variables have been shown in Table 3. The Reynolds number (Re) for the melt convection in all cases is far greater than 10,000, indicating that the flow is fully developed turbulence, and it increases with the size of the molten pool. The Richardson number (Ri) represents the importance of natural convection relative to forced convection. Calculations show that Ri ranges from 0.1 to 10, suggesting that, while the Lorentz force is dominant, buoyancy also plays a significant role, especially as the molten pool size increases. It is important to note that Ri does not consistently increase with the molten pool size; it initially decreases and then increases. This is because the phase current $I_{A}$ in the medium-scale molten pool is greater (14,000 A) than that in the small-scale molten pool (10,000 A), and the increase in Lorentz force surpasses the effect of the pool’s size increase. The Prandtl number is approximately 0.81, indicating that the thickness of the flow boundary layer and the thermal boundary layer are roughly equal. Based on the momentum boundary layer thickness formula, this thickness is estimated to range from about 1 to 2 mm.

#### 4.2.5. Thermal Balance Analysis

**Figure 13:**
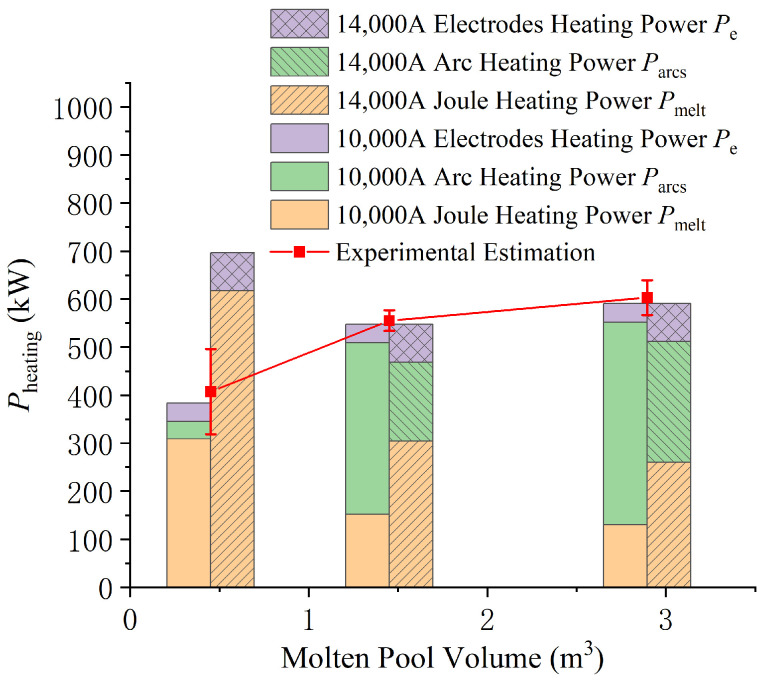
Variation in heating power ($P_{heating}$) with the volume of the molten pool: The total heating power required for different molten pool volumes (0.45 $m^{3}$, 1.45 $m^{3}$, and 2.9 $m^{3}$) closely matches experimentally estimated values. The agreement between experimental and model-calculated power highlights the model’s accuracy. This suggests that maintaining thermal balance with minimal power input after smelting can aid impurity removal and crystal growth.

The power required for an EFMF to reach a thermal balance state mainly depends on the volume of the molten pool. When the volume of the molten pool is small (0.45 $m^{3}$), medium (1.45 $m^{3}$), and large (2.9 $m^{3}$), the corresponding total heating powers required are 387 kW, 548 kW, and 591 kW, respectively, as shown in Figure 13. When the molten pool is small, a current of only 10,000 A is needed to reach thermal balance. If the current reaches 14,000 A, the total heating power will approach 700 kW (about 20% of rated power), exceeding the 387 kW required for thermal balance. When the molten pool reaches a medium or large scale, if the current is small (for example, only 10,000 A), the total power can reach the value required for thermal balance by increasing the voltage to increase the arc power.

The total power required to reach balance can be estimated using the following power conservation formula:

(14) $$P_{heating}=P_{t}-P_{newmelt}-P_{newsolid}-P_{loss}$$

where $P_{newmelt}$ is the power absorbed by the molten raw material. $P_{newsolid}$ is the power absorbed by the unmelted raw material, $P_{loss}$ is the power lost in the short network and other components, and $P_{heating}$ is the heating power required to maintain the current size of the molten pool, which is equal to the heat dissipation power, $Q_{dissipation}$.

(15) $$P_{newmelt}=\dot{m}\left(\int_{T_{0}}^{T_{m}}C_{p}\left(T\right)dT+L_{m}\right)$$

where $\dot{m}$ is melting rate, $L_{m}$ is latent heat of fusion per unit mass, $T_{m}$ is the melting point of MgO, and $T_{0}$ is the temperature of the environment.

(16) $$\dot{m}=\frac{\Delta WM}{W\Delta t}$$

where Δt is the time interval, ΔW is the electricity consumption over time period Δt, *W* is the total electricity consumption, and *M* is the total weight of the molten ingot. The above data can all be obtained through the experiments.

(17) $$P_{newsolid}=\left({\dot{m}}_{feeding}-\dot{m}\right)\int_{T_{0}}^{T_{av}}C_{p}\left(T\right)dT$$

where ${\dot{m}}_{feeding}$ is feeding rate which is monitored during the experiments, and $T_{av}$ is the average temperature of unmelted MgO.

(18) $$Q_{dissipation}=Q_{rad}+Q_{conv}$$

where $Q_{rad}$ represents radiative heat dissipation, and $Q_{conv}$ represents convective heat dissipation.

$P_{t}$ is the total input active power, which can be expressed in terms of apparent power *S* and power factor cosφ.

(19) $$P_{t}=S\times \cos\varphi =\sqrt{3}U_{L}I_{L}\cos\varphi$$

where $U_{L}$ and $I_{L}$ represent the line voltage and line current, respectively, and they are measurable parameters in the experiment. Due to the three-phase imbalance problem, the average values of phases A, B, and C are taken, respectively.

$P_{heating}$ is derived from the average value of four smelting experiments. As can be seen from Figure 13, when the scale of the molten pool is small, the fluctuation of $P_{heating}$ is relatively large. This is because there are significant differences in the processes during the initial stage of smelting. Specifically, it includes three aspects in this stage: first, the power factor fluctuates significantly; second, the consumption of graphite for ignition is relatively high; third, the current and voltage fluctuate greatly. During the middle and later stages of smelting, the fluctuation of is relatively small. Overall, the calculation results of the simulation model are in good agreement with the experimental estimated values.

### 4.3. Directions of Future Research

There are still numerous issues to be addressed regarding the smelting principles of EFMFs. Specifically, a transient model is required to address the phase-change process. The behavior of the arcs within the furnace also needs to be estimated using various models to provide a more accurate mass and heat transfer model. Material properties should be measured through low-cost experiments to verify the rationality of theoretical values.

## 5. Conclusions

(1) This paper presents a simple and effective method for analyzing the steady-state multi-physical fields of an EFME. The computational workload for calculating the temperature field and flow field is only one-sixth of that of the traditional model.

(2) A lumped-parameter circuit model is adopted to simplify the results of the electromagnetic field distribution, revealing the relationship between the shape of the molten pool and impedance characteristics. The equivalent resistance of the molten pool decreases as the molten pool expands, and it is approximately inversely proportional to the cross-sectional area of the molten pool through which the current flows.

(3) The melt undergoes extremely intense convection under the stirring of the Lorentz force, which significantly affects the overall temperature field inside EFMF.

(4) When the molten pool is small, this resistance value can provide sufficient Joule heating power for thermal balance. When the molten pool reaches a certain size, it is necessary to increase the output voltage of the transformer, thereby increasing the arc power to achieve thermal balance.

(5) The proportions of radiant and convective heat dissipation also vary with the size of the molten pool. When the molten pool is small, convective heat dissipation is dominant; when the molten pool is large, radiant heat dissipation becomes more significant.

(6) The power required for thermal balance estimated by experiments is also in good agreement with the calculation results of the model. This means that, after the smelting process is completed, if the feeding of raw materials is stopped and a relatively small amount of power is continuously input to maintain thermal balance, it may contribute to impurity removal and crystal growth.

(7) In order to address the limitations inherent in the current study, future research endeavors should place emphasis on the incorporation of transient analysis. Transient analysis is capable of accounting for the temporal variations in system variables, thus presenting a more accurate and realistic representation of the phase transition process.

## Figures and Tables

**Table 1:** Material properties of MgO and ultra-high power graphite electrodes [28,29].

Property	Values	Units
Conductivity (MgO)	3300	S/m
Relative permeability (MgO)	1	—
Conductivity (graphite electrodes)	117,647.1	S/m
Relative permeability (graphite electrodes)	1	—

**Table 2:** Dimensional Parameters of EFMF.

	Dimensions (mm)		
**Parameters**	**Large Scale**	**Medium Scale**	**Small Scale**
Charge height ($H_{f}$)		2160	
Charge radius ($R_{f}$)		1500	
Electrode center-to-center distance ($D_{e}$)		1150	
Electrode radius ($R_{el}$)		200	
Electrode length ($L_{e}$)		1600	
Pool major radius ($R_{b}$)	1137	1068	976
Pool minor radius ($R_{s}$)	918	743	463
Pool height ($H_{p}$)	1042	659	308

**Table 3:** Material properties of MgO [28,29].

Property	Values	Units
Density (ρ)	2600	kg/m^3^
Coefficient of thermal expansion (α)	0.0002	—
Melting point	3098	K
Heat capacity ($C_{p}$)	1505	J/(kg·K)
Thermal conductivity (λ)	2.6	W/(m·K)
Viscosity (μ)	$-1.5686\times 10^{-6}T+0.00626$	Pa·s
Convective heat transfer coefficient (*h*)	10	W/(m^2^·K)
Emissivity (ϵ)	0.8	—

**Table 4:** The variation in $R_{p}$, jωL, $U_{A}$, and $P_{melt}$ with the size of molten pool ($I_{A}$ = 14,000 A).

Pool Dimensions	$R_{p}$ (mΩ)	jωL (mΩ)	$U_{A}$ (V)	$P_{\mathrm{melt}}$ (kW)
Large scale	0.442	0.232	6.19	256
Medium scale	0.511	0.231	7.15	300
Small scale	1.025	0.231	14.35	603

**Table 5:** Comparison of dimensionless numbers of different molten pools.

Parameters	Definition	Small Scale	Medium Scale	Large Scale
Typical length (mm)	$L=(R_{b}+R_{s}+H_{p})/3$	560	808	1005
Average flow velocity (m/s)	$\bar{v}$	0.1	0.11	0.11
Typical temperature difference (K)	ΔT	15	9	13
Reynolds number	Re=ρvL/μ	$1.03\times 10^{5}$	$1.64\times 10^{5}$	$2.04\times 10^{5}$
Grashof number	$Gr=g\alpha \Delta TL^{3}\rho^{2}/\mu^{2}$	$1.76\times 10^{10}$	$3.67\times 10^{10}$	$8.79\times 10^{10}$
Richardson number	$Ri=Gr/Re^{2}$	1.65	1.18	2.12
Prandtl number	$Pr=C_{p}\mu /\lambda$	0.81	0.81	0.81

## Data Availability

The raw data supporting the conclusions of this article will be made available by the authors on request.

## Abbreviations and Nomenclature

### Nomenclature

**Symbol**	**Description**	**Unit**
$\dot{B}$	Magnetic flux density	T
$C_{p}$	Specific heat	J/(kg· K)
$\dot{D}$	Electric displacement	C/$m^{2}$
$D_{e}$	Electrode center-to-center distance	m
$\dot{E}$	Electric field density	N/C
$f_{L}$	Lorentz force density	N/$m^{3}$
$G_{k}$	Generation of turbulence kinetic energy	W/$m^{3}$
g	Acceleration of gravity	m/$s^{2}$
$\dot{H}$	Magnetic field strength	A/m
$H_{f}$	Charge height	m
$H_{p}$	Pool height	m
*h*	Convective heat transfer coefficient	W/($m^{2}\cdot$ K)
${\dot{I}}_{A}$	Equivalent phase current of the molten pool in phase A	A
$I_{L}$	Line current	A
$\dot{J}$	Current density	A/$m^{2}$
*k*	Turbulence kinetic energy	$m^{2}$/$s^{2}$
*L*	Typical length of the molten pool	m
$L_{e}$	Electrode length	m
$L_{m}$	Latent heat of fusion per unit mass	J/kg
*M*	Total weight of the molten ingot	kg
$\dot{m}$	Melting rate	kg/s
${\dot{m}}_{feeding}$	Feeding rate which is monitored during the experiments	kg/s
$P_{e}$	Joule heating power of all electrodes	W
$P_{melt}$	Active power absorbed by the entire molten pool	W
$P_{t}$	Total input active power	W
$P_{arcs}$	Total power released by the electric arcs	W
$P_{newmelt}$	Power absorbed by the molten raw material	W
$P_{newsolid}$	Power absorbed by the unmelted raw material	W
$P_{loss}$	Power lost in the short network and other components	W
$P_{heating}$	Total heating power released by arcs, molten pool, and electrodes	W
*Q*	Volumetric heat source	W/$m^{3}$
$Q_{Joule}$	Joule heating source	W/$m^{3}$
$Q_{arcs}$	Arc heating source	W/$m^{3}$
$Q_{dissipation}$	Heat dissipation power	W
$Q_{rad}$	Radiative heat dissipation power	W
$Q_{conv}$	Convective heat dissipation power	W
$q_{0}$	Total power released by the arcs	W
$R_{e}$	Resistance of one electrode	Ω
$R_{el}$	Electrode radius	m
$R_{b}$	Pool major radius	m
$R_{f}$	Charge radius	m
$R_{s}$	Pool minor radius	m
$R_{p}$	Equivalent resistance of the molten pool	Ω
*S*	Apparent power	VA
*T*	Temperature	K
$T_{m}$	Melting point of MgO	K
$T_{0}$	Temperature of the environment	K
$T_{av}$	Average temperature of unmelted MgO	K
*t*	Physical time	s
Δt	Time interval	s
${\dot{U}}_{A}$	Equivalent phase voltage of the molten pool in phase A	V
$U_{L}$	Line voltage	V
v	Velocity of the melt	m/s
$\bar{v}$	Average flow velocity	m/s
*W*	Total electricity consumption	J
ΔW	Electricity consumption over time period Δt	J
Gr	Grashof number
Re	Reynolds number
Ri	Richardson number
Pr	Prandtl numbere
**Greek Symbol**	**Description**	**Unit**
α	Coefficient of thermal expansion	–
ϵ	Emissivity	–
ε	Turbulent dissipation rate	$m^{2}$/$s^{3}$
cosφ	Power factor	–
λ	Thermal conductivity	W/(m·K)
μ	Viscosity	Pas
ρ	Density	kg/$m^{3}$
σ	Electrical conductivity	S/m
ω	Angular frequency	rad/s
